# Electro-Optic Response of Polymer-Stabilized Cholesteric Liquid Crystals with Different Polymer Concentrations

**DOI:** 10.3390/polym16172436

**Published:** 2024-08-28

**Authors:** Lotfi Saadaoui, Donghao Yang, Faheem Hassan, Ziyang Qiu, Yu Wang, Yujie Fan, Irena Drevensek-Olenik, Yigang Li, Xinzheng Zhang, Jingjun Xu

**Affiliations:** 1 The MOE Key Laboratory of Weak-Light Nonlinear Photonics and International Sino-Slovenian Join Research Center on Liquid Crystal Photonics, TEDA Institute of Applied Physics and School of Physics, Nankai University, Tianjin 300457, China; lotfi.saadaoui@fst.utm.tn (L.S.); yangdonghao0305@126.com (D.Y.); faheemhassan118@hotmail.com (F.H.); 2120230309@mail.nankai.edu.cn (Z.Q.); 1120190080@mail.nankai.edu.cn (Y.W.); 2210369@mail.nankai.edu.cn (Y.F.); liyigang@nankai.edu.cn (Y.L.); jjxu@nankai.edu.cn (J.X.); 2 Physics Laboratory of Soft Matter and Electromagnetic Modelling, Faculty of Sciences of Tunis, University of Tunis El Manar, El Manar Tunis 2092, Tunisia; 3 Faculty of Mathematics and Physics, University of Ljubljana, SI-1000 Ljubljana, Slovenia; 4 Department of Complex Matter, J. Stefan Institute, SI-1000 Ljubljana, Slovenia; 5 Collaborative Innovation Center of Extreme Optics, Shanxi University, Taiyuan 030006, China

**Keywords:** liquid crystals, polymer-stabilized, lasing emission, dissymmetry factor

## Abstract

Polymer-stabilized cholesteric liquid crystals (PSCLCs) have emerged as promising candidates for one-dimensional photonic lattices that enable precise tuning of the photonic band gap (PBG). This work systematically investigates the effect of polymer concentrations on the AC electric field-induced tuning of the PBG in PSCLCs, in so doing it explores a range of concentrations and provides new insights into how polymer concentration affects both the stabilization of cholesteric textures and the electro-optic response. We demonstrate that low polymer concentrations (≈3 wt. %) cause a blue shift in the short wavelength band edge, while high concentrations (≈10 wt. %) lead to a contraction and deterioration of the reflection band. Polarization optical microscopy was conducted to confirm the phase transition induced by the application of an electric field. The observations confirm that increased polymer concentration stabilizes the cholesteric texture. Particularly, the highly desired fingerprint texture was stabilized in a sample with 10 wt. % of the polymer, whereas it was unstable for lower polymer concentrations. Additionally, higher polymer concentrations also improved the dissymmetry factor and stability of the lasing emission, with the dissymmetry factor reaching the value of around 2 for samples with 10 wt. % of polymer additive. Our results provide valuable comprehension into the design of advanced PSCLC structures with tunable optical properties, enhancing device performance and paving the way for innovative photonic applications.

## 1. Introduction

Research on photonic band structure has significantly enhanced our knowledge of how light behaves in periodic dielectric materials, particularly when the periodicity is comparable to the wavelength of light. For example, photonic band gaps (PBGs) in photonic crystals (PCs) play a crucial role in controlling light propagation by preventing its propagation within a specific frequency region. The propagation of light is completely forbidden inside the PBG, while it is enhanced at the edges [1,2,3]. Among the diverse range of PCs, photonic liquid crystals stand out due to their intricate structural complexity and unique optical properties, including their distinctive sensitivity to light polarization. Cholesteric liquid crystals (CLCs) act as one-dimensional photonic crystals, characterized by a sufficiently strong periodic modulation of the refractive index [3,4]. CLCs are formed by a class of molecules capable of self-organizing into helical structures and exhibiting distinctive circularly polarized reflection properties [5,6]. The length over which the liquid crystal (LC) directors rotate by 2π is defined as helical pitch (*p*). Circularly polarized light (CPL) that matches the handedness of the helix is reflected, whereas CPL with a handedness opposite to that of the helix is transmitted [5]. This inherent ability for spontaneous assembly, coupled with their optical characteristics, makes them a highly promising technology across various sectors. Their applications span from reflective displays to tunable mirrorless lasers, optical storage solutions, adjustable color filters, and intelligent windows [7,8,9].

Thus, the helical arrangement of CLCs can be effectively maintained through the in situ photopolymerization of liquid crystal monomers within CLC mixtures, leading to the formation of polymer-stabilized CLCs (PSCLCs) [10,11,12]. Meanwhile, the polymer network remains unchanged, providing a restoring force that promotes stable textures [13]. PSCLCs offer a responsive optical behavior that can be triggered by external factors such as electric fields, thermal stimuli, or light exposure [11,14,15,16]. In most PSCLC-based electro-optical devices, the orientation of CLC molecules is altered by externally applied electric fields, thereby modifying the optical properties of the CLC cell [13]. This dynamic nature enables fine-tuning of their optical properties, rendering them adaptable for diverse applications spanning from photonics to displays, sensing technologies, laser, and more [16,17,18,19,20,21]. In recent years, PSCLCs have emerged as promising candidates for achieving targeted tuning within specific regions of the PBG, making them good choices for fabricating electro-tunable laser devices [10,20,22]. As we know, lasing from CLCs occurs more probably at the long wavelength band-edge (LWBE) than the short wavelength band-edge (SWBE) of the PBG due to the high density of the photon state and the effective alignment of the dipole moment of the laser dye molecules with the local director of the liquid crystal [3,23,24,25,26]. Consequently, electrically shifting LBEW of the reflection band in CLC offers an easy way to tune the lasing emission wavelength. Tuning the PBG width while keeping the effective birefringence of the LC relatively constant can be accomplished by (i) introducing spatial variations in the helical pitch across the cell gap under the presence of external stimuli or by (ii) modulation of the refractive index. Various methods have been realized using the spatial variation in the helical pitch, which represents the most popular method used to tune the PBG width, but they often exhibit slow response times, typically in the range of seconds. For instance, Hu et al. showed that CLCs with negative dielectric anisotropy and chiral ionic liquids composite could be switched electrically between a transparent, scattering, and reflecting state in the visible region [27]. Upon the initial application of a DC electric field and then of an AC electric field, the bandwidth of the reflection band can be controlled accurately and reversibly by adjusting the intensity of the DC electric field. Similarly, the broadening of a PSCLC reflection band gap under the application of the DC field has been reported by Tondiglia et al. [28]. They reported a dramatically different electro-optical effect, one that occurred in PSCLC cells with small net positive or negative dielectric anisotropy in homogeneously aligned cell configurations. The application of weak DC electric fields was found to result in large symmetric increases in the reflection stop band. On the other hand, a limited body of research has explored the electric-field-induced PBG broadening using modulation of the refractive index. Thus, Ozaki et al. demonstrated a deformation-free microsecond electro-optic tuning of PSCLCS with negative dielectric anisotropy [29], which was achieved by embedding a small droplet of liquid crystals in a polymerized liquid crystal matrix.

In this work, electrically tunable optical characteristics of PSCLC composite systems with positive dielectric anisotropy were studied. Chiral polymer composite systems were produced through the photopolymerization of a small quantity (≤10%) of reactive mesogenic monomers into cholesteric liquid crystals. Optical measurements were conducted to assess the effect of the polymer concentration on the PBG tuning of three PSCLC samples under electric field excitation. Furthermore, the degree of the circular polarization of luminescence from a dye-doped PSCLC was conducted. In addition, electrically tunable lasing emission from the dye-doped PSCLC samples was demonstrated. The results show that the high polymer concentration (10 wt. %) typically enables the appearance of more stable cholesteric phases, increases the threshold electric field for each transition and improves the dissymmetry factor *g*(*λ*). CPL spectra show that *g*(*λ*) values increase from 1.6 to 1.98 when the polymer concentration changes from 3 wt. % to 10 wt. %. Consequently, we found that laser intensity remained unchanged in samples with high polymer concentrations even at strong electric fields and that the emission wavelength could be adjusted electrically. However, in samples with low polymer concentrations, the lasing intensity decreased when applying a weak electric field. We attributed this effect to the modulation of the refractive index, which originated from the local reorientation of the LC molecules dispersed in the polymer matrix. These findings show a notable feature of PSCLCs: their ability to selectively shift the lasing emission wavelength within the short and/or the long reflection band edges of a CLC. 

Previous studies have established that variations in polymer concentration can influence the stability and optical properties of PSCLCs. However, our research delves deeper into the specific effects on PBG tuning and phase transitions. Unlike earlier studies that have primarily focused on broader electro-optic responses, this research specifically examines the impact of polymer concentrations on the PBG and cholesteric texture stability under an AC electric field. Additionally, we focus on the controlled tuning of the PBG and the stabilization of the cholesteric texture, particularly the fingerprint texture, at specific polymer concentrations. Overall, this work underscores the significance of investigating the effects of polymer concentrations in PSCLCs. For future research, a promising direction would be to explore the effects of new kinds of chiral agent [30], different monomers, cross-linkers, or liquid crystal compounds to achieve even finer control over the photonic band gap, texture stability, and overall device performance. This could offer additional perspectives on tuning the electro-optic properties and further enhancing the performance of PSCLCs.

## 2. Experimental Section

### 2.1. Sample Preparation

Cholesteric liquid crystals were prepared by mixing a nematic liquid crystal QYTN-009 and a right-handed chiral dopant R5011 with a high helical twisting power of 116 µm^−1^ at 20 °C, both from Qingdao QY Liquid Crystal Co., Ltd., Qingdao, China. The extraordinary and ordinary refractive indices of QYTN-009 are *n_e_* = 1.675 and *n_o_* = 1.516 at *T* = 20 °C and *λ* = 589 nm, respectively. For the PSCLC mixtures, an achiral monomer RM257, *n_e_* = 1.687 and *n_o_* = 1.508 at *T* = 20 °C and *λ* = 589 nm was added to the CLC mixtures [31]. Three PSCLCS samples were prepared (S1, S2, and S3) with different weight ratios. The nematic liquid crystal QYTN-009 and the chiral dopant R5011 were chosen for their well-defined refractive indices and high helical twisting power, which are crucial for achieving the desired cholesteric phase at room temperature and precise control over the PBG. The achiral monomer RM257 was selected for its ability to polymerize effectively within the cholesteric matrix, providing structural stability without disrupting the liquid crystal’s optical properties. The refractive indices of RM257 closely match those of the liquid crystal, minimizing refractive index mismatches and ensuring uniform alignment. These qualities make these materials ideal for studying the tunability of PSCLCs, particularly in understanding how polymer concentration influences the electro-optic response and texture stability.

**Figure 1:**
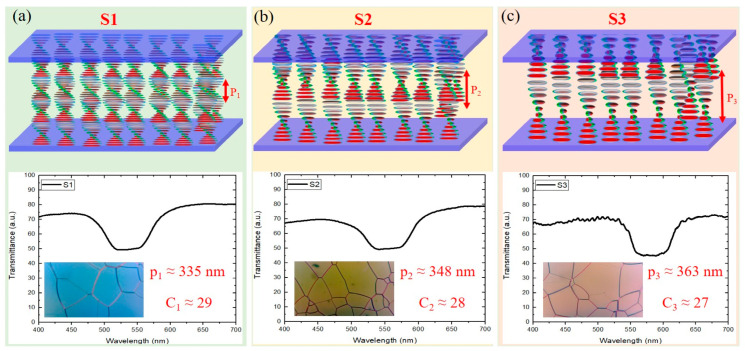
(Upper panels) Schematic illustration of the three PSCLC samples: (**a**) S1 (3 wt. %), (**b**) S2 (6 wt. %), and (**c**) S3 (10 wt. %); (lower panels) the PBG spectra for each sample, along with their corresponding microscopic observations.

Our study specifically explores the electro-optical properties at concentrations less than 10 wt%, to investigate the behavior of PSCLCs at lower polymer concentrations. We selected three specific concentrations, 3 wt. %, 6 wt. %, and 10 wt. %, to explore the electro-optical properties across a range of lower concentrations. Although the choice of these particular concentrations was somewhat arbitrary, it was guided by our interest in understanding how varying polymer levels influence the stabilization of the cholesteric texture and the tunability of the photonic band gap. This approach allows us to gain insights into how low concentrations influence the stabilization of the cholesteric texture and the tunability of the photonic band gap. Table 1 illustrates the composition and the different weight concentrations of the three PCLC samples:

The photoinitiator IRG651 was added into the prepared mixtures to assist the photopolymerization and stabilize the PSCLC mixtures. The mixtures were in a cholesteric phase at room temperature with a distinctive reflection band. Then, the mixtures were filled into 10 μm-thick cells consisting of two parallel glass substrates with ITO coating (electrode). To obtain the planar orientation of the CLC molecules, the inner surfaces of the glass plates were coated with polyvinyl alcohol (PVA) and then rubbed in the antiparallel directions. PVA was dissolved in water at a concentration of 0.5% in weight. The PVA coating procedure was carried out as follows: The glass substrate was first secured onto a rotating disk using air suction. A few drops of the PVA solution were then carefully applied to the substrate with a pipette. The substrate was spun at 3000 rpm for 60 s, forming a uniform PVA layer approximately 50 nm thick. The resulting mixture was heated in isotropic phase to 90 °C, and then driven with a magnetic stirrer at a rotation speed of 1000 rpm and a rotation time of 10 min. The cells were cooled from 90 °C to room temperature and confirmed that a monodomain Grandjean planar texture was obtained. Then, the cells were illuminated with a UV light source with an output power density equal to 20 mW/cm^2^ for 5 min on each side at a wavelength of 365 nm to complete the photo-polymerization of the whole mixture. During the photo-polymerization process, the reactive mesogen phase separated from the liquid crystals to form polymer networks, which mimicked the helical structure of the liquid crystal [32]. The cross-linked polymer network acts as an anchoring framework, limiting the mobility of the LC molecules. This anchoring can stabilize the cholesteric helix, leading to enhanced texture stability and altering the LC’s response to electric fields. In addition, due to the proximity of the polymer to the liquid crystal molecules, the LC molecules can form weak interactions, such as van der Waals forces or hydrogen bonding, with the polymer chains. These interactions can influence the local alignment of LC molecules, affecting the overall electro-optic properties of the PSCLC. Figure 1 illustrates the schematic sketch of the three PSCLC samples without any bias. S1, S2, and S3 exhibit a PBG spanning 553–519 nm, 580–537 nm, and 601–558 nm with a full width of ~34 nm, ~43 nm, and ~43 nm, respectively. Thus, the calculated pitch lengths of the three PCLC samples are *p*_1_ = 335 nm, *p*_2_ = 348 nm, and *p*_3_ = 363 nm, respectively, resulting in a high confinement ratio *C_i_*_=1,2,3_ = *d*/*p* >> 1, where *C* is defined as the ratio of the cell thickness over the cholesteric natural pitch.

### 2.2. Transmission Spectra Measurement to Characterize the Reflection Band Gap

**Figure 2:**
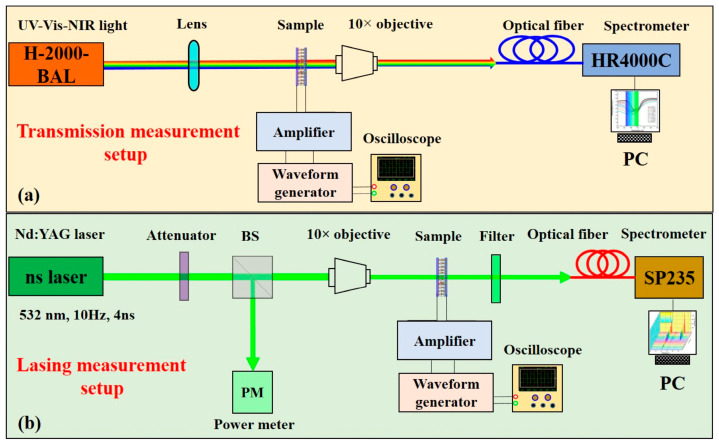
(**a**) Experimental setup to measure the spectral response of the pure CLC and PCLC samples and (**b**) experimental setup to test the lasing performance.

To record the spectral response of each sample and its photonic band edge during the application of the electric field, non-polarized white halogen light from a UV-Vis-NIR light source (DH-2000-BAL, Ocean Optics, Duiven, The Netherlands), was focused onto the samples at normal incidence. Figure 2a illustrates the experimental setup used in our study to analyze the spectral response of the pure CLC and PCLC mixtures and their photonic band edges. To focus the incident light on the cell to a spot size with diameter of 0.5 mm, a lens with a focal length of 10 cm was placed before the sample. The emitted light from the sample was collected by a 10× microscope objective (NA = 0.25) and then transmitted via an optical fiber (core diameter = 200 μm) to the high-resolution (0.5 nm) spectrometer (HR4000CG-UV-NIR, Ocean Optics, Duiven, The Netherlands). Then, transmission spectra were collected by an optical fiber (core diameter = 200 μm) coupled to a high-resolution spectrometer (0.5 nm), HR4000CG-UV-NIR (Ocean Optics). An optical polarizing microscope (POM) was used to observe the cholesteric texture and its modification under the application of the AC field. In addition, POM observation was conducted to approve the threshold E-field for the cholesteric texture transformation.

### 2.3. Application of an Electric Field

A square-wave electric field was applied across the glass cells using a waveform generator (Agilent 33120A, Boulder, CO, USA) operating at a frequency of 1 kHz connected to a high-voltage amplifier (NF3211, Newport Opto-Electronics Technologies Co., Ltd., Wuxi, China). The signals from the amplifier were closely monitored using a digitizing oscilloscope (HP54602B, TECOO Technology Co., Ltd., Shenzhen, China) to ensure that the waveform remained undistorted even at high voltage.

### 2.4. Optical Pumping

For lasing experiments, the second harmonic of a Q-switched Nd: YAG laser (Continuum, Surelite III) was used as a pumping light source. The pulse wavelength, width, and repetition rate were 532 nm, 4 ns, and 1 Hz, respectively. The laser beam was attenuated and then focused by a 10× microscope objective (NA = 0.28) to reduce the spot of the laser beam on the cholesteric cell to a few hundred micrometers. The beam was divided into two beams, and the pulse energy was monitored with an energy meter (LabMax-Top, Coherent Inc., Saxonburg, PA, USA) in the reflected beam. The transmitted pump beam irradiated in a manner perpendicular to the sample, whose intensity was controlled using neutral density filters. An optical fiber (model BF F-455-7, core diameter = 200 µm, Princeton, NJ, USA) was coupled to a high-resolution (0.14 nm) spectrometer (SP2358, Princeton Instruments, Trenton, NJ, USA) to collect the light emitted from the sample. Figure 2b shows the optical setup to test the lasing performance of the two cells. It is worth noting that the measurements were carried out under ambient conditions at a standard room temperature of 25 °C and to guarantee the repeatability and reliability of our results, we implemented several crucial measures such as conducting tests on multiple batches, repeating experiments, and calibrating the equipment.

## 3. Results and Discussion

### 3.1. Wavelength Tuning of the PBG in PSCLC Composites

**Figure 3:**
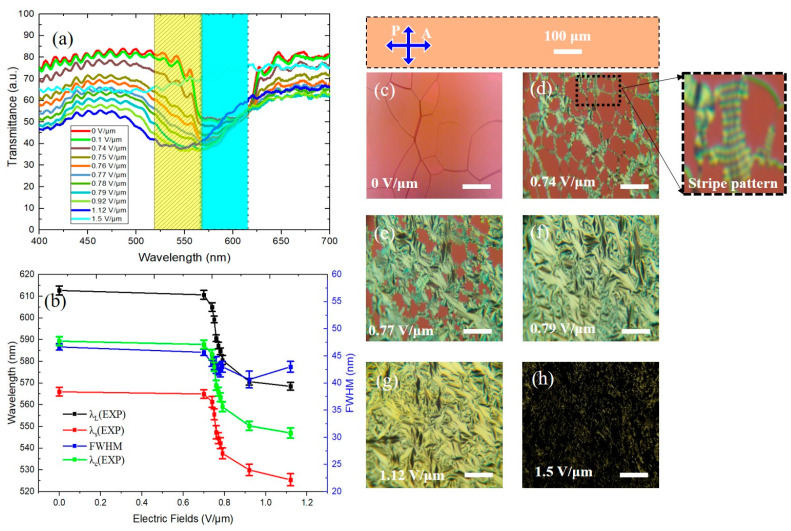
(**a**) The transmission spectra showing the blue shift of the PBG as an E-field is applied to the pure CLC sample (without reactive mesogen). (**b**) Plots of the LWBE (λ_L_(EXP)), SWBE (λ_s_(EXP)) wavelengths (black and red curve), central wavelength (λ_c_(EXP): green curve), and FWHM (blue curve) as a function of the applied electric field, the error bars indicate the standard deviation. The blue and yellow rectangles show the position of the PBG at zero field and 1.5 V/µm respectively. (**c**–**h**) POM images showing the birefringence change of the planar texture. The scale bars represent 100 μm.

Initially, to understand the impact of the polymer concentration in the three PSCLC samples on the resulting range of wavelength tuning in the band gap under AC voltage, a pure CLC sample with 2.4 wt. % of R5011 doped into 97.6 wt. % of QYTN009 was prepared and investigated. The transmission spectra and corresponding POM images for various electric field amplitudes are shown in Figure 3. At zero field, the cholesteric sample exhibits a reflection band ranging from 613 nm to 565 nm, corresponding to the LWBE and the SWBE, respectively. The POM observation highlights the presence of oily streak defects, which are typical characteristic patterns associated with the planar state (Figure 3c). These defects arise from localized disturbances in the orientation of LC molecules within the planar alignment. At relatively low electric fields, a noticeable blue shift occurs in the PBG, as depicted in Figure 3a. This shift in the PBG is generally attributed to a transition in the cholesteric texture, as evidenced by the POM observations (Figure 3c–h). For an E-field of 0.74 V/μm, a change in the color of the optical texture was observed, indicating the transition of the planar cholesteric state to the well-known fingerprint texture (Figure 3d). As we know, when an external electric field is applied perpendicular to the plane of the CLC cell, it disrupts the uniform alignment of the LC molecules, causing them to tilt toward the electric field direction. Simultaneously, the LC molecules strive to maintain their helical structure because of intermolecular interactions. This competition between aligning with the electric field and preserving the helical structure results in the liquid crystal layers tilting and forming finger-like structures. These structures undulate in a sinusoidal or zigzag stripe pattern along the cell surface, as shown in inset Figure 3d. The stripe direction depends on the confinement ratio *C* of the cell gap to the cholesteric pitch length [33]. The Helfrich–Hurault instability is known to be dedicated to the formation of these undulation structures, in which the layer of the helix is undulated in the cell-plane direction [34,35]. As the electric field increases, the fingerprint texture collapses into the focal conic state (Figure 3e). Usually, the fingerprint texture in a pure CLC is difficult to hold under electric fields and immediately undergoes a transition to focal conic texture. In this case, the time required to transition from the fingerprint state to the focal conic state is relatively fast, on the order of milliseconds, making this transition observable only in a tiny voltage range. In the focal conic state, (Figure 3f,g), the helical structure is arbitrarily arranged. At a threshold E-field, the helical structure starts to unwind between the cell boundaries and the focal conic state collapses to the homeotropic state (Figure 3h). The unwinding electric field (*E*_unw_) is defined as the threshold field at which the homeotropic state starts to form, which is found to be around 1.5 V/μm. In the homeotropic state, the helical structure is distorted, the LC molecules align parallel to the electric field and the shape of the PBG is suppressed. This transition is marked by a change in the color structure to a dark appearance [33]. In more technical terms, the observed decrease in wavelength in the pure sample when increasing the electric field could be explained by the influence of the field on the positive liquid crystal. When an electric field is applied, it exerts a torque on the LC molecules, causing them to re-align along the direction of the field. This realignment leads to a change in the effective refractive index of the LC mixture. Specifically, as the electric field strength increases, the average refractive index of the LC decreases. This decrease in the refractive index, in turn, reduces the wavelength of the reflected light, shifting the PBG to shorter wavelengths (a blue shift).

To extract the values of LWBE and SWBE from the experimental spectra, we employed the Berreman 4 × 4 matrix method to fit the experimental data. By adjusting the theoretical transmission spectra to match the experimental spectra through variations in the number of the cholesteric layers (N) and the pitch (p), we could determine the values of the long and short edges of the PBG. The SWBE and LWBE were determined using the equations *λ_LWBE_* = *ne*·*p* and *λ_SWBE_* = *no*·*p*. The computed results were consistent with our observations. In Figure S1, we illustrate both the experimental results extracted directly from the spectrum and the fitted results as well as the variation of the LWBE, SWBE, central wavelength, and the width of the PBG.

Figure 3b shows the LWBE, SWBE (black and red curves), and the central wavelength (green curve) as a function of the applied electric field. With increasing field strength, both the SWBE and LWBE shift simultaneously, with a modification of the helical pitch. Remarkably, the LWBE and the SWBE shift from 612 nm and 566 nm to 568 nm and 525 nm, respectively (corresponding to a total shift of Δ*λ_LWBE_* ≈ 45 nm), and therefore the full width at half maximum (FWHM) of the band gap is found to be ~46 nm.

**Figure 4:**
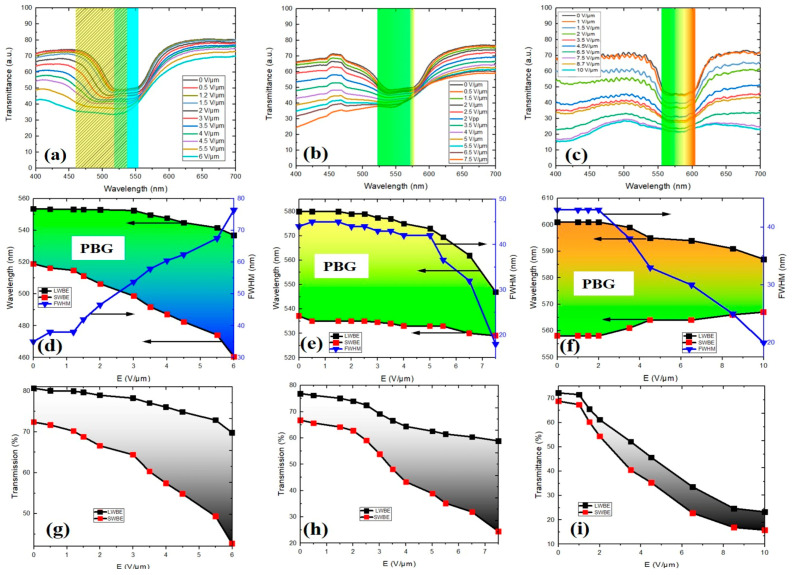
Transmission spectra for samples (**a**) S1, (**b**) S2, and (**c**) S3. The changes of *λ_LWBE_*, *λ_SWBE_* and FWHM with the applied voltages for (**d**) S1, (**e**) S2, and (**f**) S3. Transparency at the LWBE (black rectangles) and SWBE (red rectangles) of the PBGs for samples (**g**) S1, (**h**) S2, and (**i**) S3.

**Figure 5:**
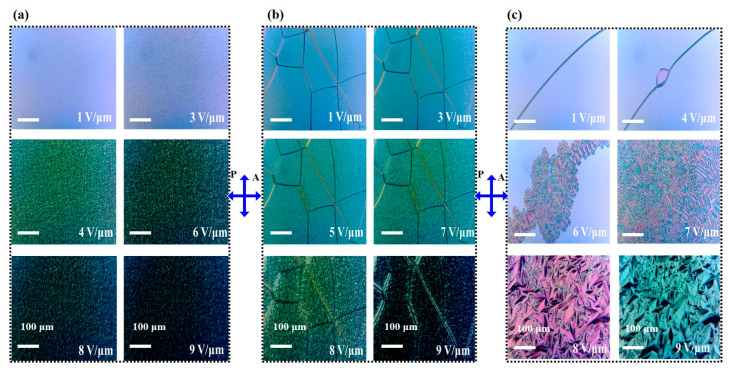
POM images showing the textures under different applied electric fields for S1 (**a**), S2 (**b**), and S3 (**c**).

In the following section, to understand the impact of the polymer concentration on the tuning range of the band gap under AC voltage, the electro-optical characteristics of the PSCLC samples were investigated. Figure 4a–c illustrate the PBG evolutions at different electric field amplitudes for the three samples S1, S2, and S3. For the three samples, at field off, the initial CLC textures are in the planar state, in which the helical axes are perpendicular to the cell surfaces, as evidenced by the presence of a monodomain Grandjean texture across the entire cell surfaces and as shown by POM micrographs (Figure 5a–c).

For sample S1, the application of an AC field resulted in an asymmetric broadening of the PBG (Figure 4a). Initially, the PBG encompassed a small portion of the green region of the visible spectrum. As the E-field increased, the PBG exhibited a blue shift. Consequently, the PBG expanded to cover a significant portion of both the green and blue regions of the visible spectrum. The SWBE shifted from 519 nm at zero field to 460 nm at 6 V/μm (red rectangles in Figure 4d), whereas the LWBE shifted from 554 nm to 537 nm (black rectangles in Figure 4d). Remarkably, the SWBE exhibited a significant blue shift of approximately 59 nm with increasing field amplitude, accompanied by reduced transmission, while the LWBE underwent a slight blue shift of 17 nm. The asymmetrical broadening of the PBG directly and significantly affected the FWHM, resulting in an increase from 35 nm at 0 V/μm to 77 nm at 6 V/μm, as depicted by the blue curve in Figure 4d. The maximum position of the PBG shifting occurred for an electric field around 6 V/μm.

At a threshold field of 4 V/μm, a two-dimensional pattern with white and black dots appeared, accompanied by a change in the color of the optical texture as recorded by the POM micrograph (Figure 5a). This pattern was responsible for the transition from the planar state texture to the fingerprint texture. For a higher electric field (*E* > 9 V/μm), the PBG was suppressed, and, as a result, the planar texture collapsed into a homeotropic state in which the LC molecule aligned with the electric field (Figure 5a). In this case, the threshold unwinding electric field *E*_unw_ was found to be around 8 V/μm. The distortion of the helical axis affected the transmission of the incident light by increasing the scattering light. Figure 4g illustrates the transmission at both SWBE and LWBE. The transmission at SWBE showed a significant decrease of ~30%, dropping from 72.4% at 0 voltage to 42.6% at 6 V/μm. In contrast, the transmission at LWBE experienced a smaller change of ~10%, decreasing from 80.7% to 69.8%. This reduction in transparency could be attributed to local molecular fluctuations caused by the electric field, which affected the orientation of the helical axis within the cell and increased light scattering. It is important to mention here that, to ensure that the observed changes in S1 were indeed due to the polymer concentration and not influenced by other variables, we prepared additional mixtures with the same polymer concentration to confirm the effects observed. The control sample exhibited similar behavior to S1.

Unlike the sample S1, the PBG of sample S2 did not exhibit any broadening, as shown by the transmission spectra in Figure 4b. Instead, the PBG exhibited a blue shift of the LWBE and a slight shift of the SWBE. As the voltage increased beyond a critical value, the LWBE underwent a blue shift, reaching 547 nm at 7.5 V/μm, with a maximum shift of 33 nm (black rectangles in Figure 4e). Under the same conditions, the SWBE skewed by approximately ~8 nm, as shown by the red rectangles in Figure 4e. This asymmetric shifting leads to a compression of the width of the PBG (blue curve, Figure 4e).

The transmission at the SWBE decreased from 66.8% to 24.5%, while the transmission at the LWBE dropped from 76.9% to 58.9% as the voltage increased from zero to 7.5 V/μm, respectively. Compared with sample S1, at field off, the oily streak defects became more pronounced and covered the entire surface (Figure 5b). This phenomenon can be attributed to the high concentration of the polymer in the mixture and to the perfect alignment of the rubbing layer, which influenced the orientation of the molecules. At an electric field of 5 V/μm, the planar state transformed to the fingerprint texture, and a change in the color of the optical texture was observed in the POM micrographs with a 2D pattern with white and black dots (Figure 5b). This is similar to what was observed in sample S1, when the E-field reached the unwinding threshold a transition from the planar state to the homeotropic state. For sample S2, *E*_unw_ was found to be around 10 V/μm, which was slightly higher than that for sample S1.

Furthermore, the PBG of S3 demonstrated distinctive features under the application of the electric field compared with S1 and S2. In contrast, when the AC field was applied in S3, there was no discernable shifting of the PBG, as evidenced by the transmission spectra in Figure 4c. There was a deterioration of the PBG, which became less sharp at both the LWBE and SWBE under AC voltage. This distortion led to a compression of the PBG width (blue curve Figure 4f). When the AC voltage further increased, the boundaries of the PBG became blurred, indicating a broader and more diffuse wavelength range over which light transmission changed (Figure 5c). This implies that the transition between high and low transmission regions at the band edges becomes more gradual and less defined. This can be attributed to the increased scattering and the change in the orientation of LC molecules in the presence of a high polymer concentration compared with S1 and S2, which affects the overall optical properties [36]. Figure 4i illustrates the transmission at LWBE and SWBE, showing a symmetric reduction of the transmission. The application of an electric field caused a decrease in the transmission at both band edges, from ~70% at zero field to ~20% at 10 V/μm, accompanied by a contraction of the PBG. This contraction can be explained by the texture deformation, as shown by microscopic observation in Figure 5c. By scanning the entire cell surface, we observed that the deformation typically began near the line defects when the electric field was applied. Therefore, we focused on a single-line defect to facilitate a more detailed investigation. For a weakly twisted CLC cell (*C* << 1), as in the studied cases, the cholesteric grating stripes appeared at the center and then spread to cover the entire cell surface. At a threshold E-field (*E_FP_* = 7 V/μm), the cholesteric undulations spread and covered the entire cell surface. As mentioned before, this texture can be attributed to the well-known CLC fingerprint texture, which is recognized as the Helfrich–Hurault deformation, as illustrated in Figure 5c. When the electric field exceeds the threshold value (*E_FC_ > E_FP_*), another structure that could emerge is the focal conic state, as illustrated in Figure 5c. In this state, the molecules were no longer in a regular helical pattern but instead formed conical or elliptical domains with varying orientations, where the helical axis was more or less randomly oriented throughout the cell. This resulted in a diffuse scattering of light, giving the texture a cloudy or speckled appearance. By increasing the E-field, two focal conic structures were obtained, namely FC1 and FC2. FC1 was obtained at a threshold field *E_FC1_* = 8 V/μm, while FC2 was observed at *E_FC2_* = 10 V/μm. Increasing the E-field (*E_unw_ > E_FC_*), the focal conic state collapsed into the homeotropic structure at *E_unw_* ~12 V/μm. After removing the E-field, the shape of the PBG could be recovered, indicating the planar texture formation. The transmittance spectra and POM micrographs at field off are illustrated in Figure S2a,b. 

### 3.2. Discussion

To explain the mechanisms responsible for the PBG tuning, let us start with sample S1, in which the SWBE exhibited a significant blue shift of approximately 60 nm with increasing E-field amplitude, while the LWBE simultaneously underwent a slight blue shift of ~16 nm. The bandwidth of the reflected light is determined by the helical pitch (*p*), the ordinary *n_o_* and extraordinary *n_e_* refractive indices, the process of which is given by the following equation: ∆*λ* = ∆*n p* = *(n_e_* − *n_o_) p*, with a central wavelength at *λ_c_* = *<n> p* for normal incidence and where <*n*> is the average refractive index of the liquid crystal, defined as <*n*> = (*n_e_* + *n_o_*)/2. The SWBE and LWBE wavelengths for the CLC can be calculated by *λ_SWBE_* = *n_o_ p* and *λ_LWBE_* = *n_e_ p*. It is known that, if the cholesteric pitch changes, both SWBE and LWBE wavelengths will change accordingly. Here, the SWBE was blue shifted while the LWBE was slightly shifted under low voltage. In comparison with existing literature, it has been reported that the shift of the SWBE typically occurs simultaneously with a shift of the LWBE. For instance, Wood et al. observed a maximum shift of 55 nm of the LWBE, accompanied by a shift of 32 nm of the SWBE in a polymer-templated sample. They attributed this additional tuning of the PBG to the reduction of the pitch of the structure as a result of a translation of the polymer fibrils [13]. They claimed that the field interaction with the liquid crystal appears to drive the potential reorganization of the polymer network. The significant blue shift of the PBG observed in sample S1, Δ*λ_SWBE_* = 60 nm and Δ*λ_LWBE_* = 16 nm, can be partially attributed to the reduction of the cholesteric pitch, but its underlying mechanisms are not yet fully understood and merit further investigation.

The sample S2 exhibited an asymmetric shifting of the LWBE side (Δ*λ_LWBE_* ≈ 28 nm) and a partial shift of the SWBE side (Δ*λ_SWBE_* ≈ 8 nm). Recently, the blue shift of *λ_LWBE_* can be attributed to a reduction in *n_e_*, while *n_o_* and *p* remain fixed. This change in *n_e_* could be attributed to the local reorientation of the LC molecules embedded in the polymer matrix. The application of an external E-field generates volumetric torques on LC molecules, which attempt to align the molecule directors with the E-field. As the orientation of the LC changes, the ordinary refractive index stays constant, while the extraordinary refractive index changes. Notably, there will be a variation of the effective refractive index, defined as *n_eff_* = *n_o_*·*n_e_*/(*n_e_*^2^ cos^2^*θ + n_o_*^2^ *s*in^2^*θ*)^1/2^, where *θ* is the angle between the helix axis and the local molecule director. Our findings agree well with those reported in the literature [13,37]. Using *λ_LWBE_* = *n_e_*·*p,* we can determine that the wavelength tuning observed for S2 corresponds to a change in *n_e_* of approximate *δn_e_* ≈ 0.09. Besides that, S3 exhibited a symmetric shifting of both the LWBE and SWBE. There was a contraction of the PBG accompanied by a deterioration of the PBG at the high E-field. This contraction can be explained by a reduction in the effective refractive index, *n_eff_.* This change in *n_eff_* can explain also the transformation of the cholesteric textures. 

The observed changes in PBGs with varying polymer concentrations could be explained through the interplay between cholesteric pitch, refractive indices, and the effective refractive index in PSCLCs. The bandwidth of the PBG in a CLC system is directly related to the effective refractive index and the helical pitch of the cholesteric structure. When the effective refractive index *n*_eff_ changes (due to the application of the electric field), it can alter the birefringence Δn and subsequently affect the bandwidth Δλ of the PBG. Specifically, (i) if the extraordinary refractive index *n*_*e*_ increases while *n*_*o*_ remains constant, the birefringence Δn increases, leading to a broader PBG (larger Δλ). (ii) If *n*_*e*_ decreases, Δn decreases, which narrows the PBG (smaller Δλ). It is important to note that the theoretical explanation could explain the behavior of samples S2 and S3 but not S1. In the former sample, we observed a blue shift of the shorter wavelength of the PBG but its mechanisms are not yet fully understood and merit further investigation.

The application of an electric field could affect the cholesteric phase by the following: (i) Reorienting the liquid crystal molecules: When an electric field is applied, it exerts a torque on the positive liquid crystal, particularly on the dipole moments of the molecules. This torque causes the molecules to reorient, typically aligning along the direction of the electric field. The degree of reorientation depends on the strength of the electric field and the dielectric anisotropy of the liquid crystal. (ii) Modulating the helical pitch: The reorientation of liquid crystal molecules under the electric field can lead to changes in the helical pitch of the cholesteric phase. A reduction in the pitch results in a blue shift of the photonic band gap (PBG), as observed in the study. (iii) Interacting with the polymer matrix to stabilize or transition the texture: The cross-linked polymer network formed during the photopolymerization process provides an anchoring effect, which resists the reorientation of the liquid crystal molecules under the influence of the electric field. This stabilization is particularly important in maintaining the alignment of the cholesteric phase and preventing disorder of the helical structure. Depending on the field strength and polymer concentration, the electric field can induce transitions between different cholesteric textures.

Table 2 illustrates the threshold electric field at different texture transitions, fingerprint (*E_FP_*), focal conic (*E_FC_*), and homeotropic (*E_H_*) states for the pure sample (S0) and the three PSCLC samples (S1, S2, and S3). The transition voltages increase with the polymer concentration. For S0, S1, and S2, the threshold E-field *E_FC_* of the planar–focal conic transition was lower than the threshold voltage V_FP_ of the fingerprint texture. While for S3, the threshold E-field *E_FP_* was lower than the threshold E-field *E_FC_*. S3 had two FC states, the first FC1 appeared at 8 V/μm and FC2 appeared at 9 V/μm.

**Figure 6:**
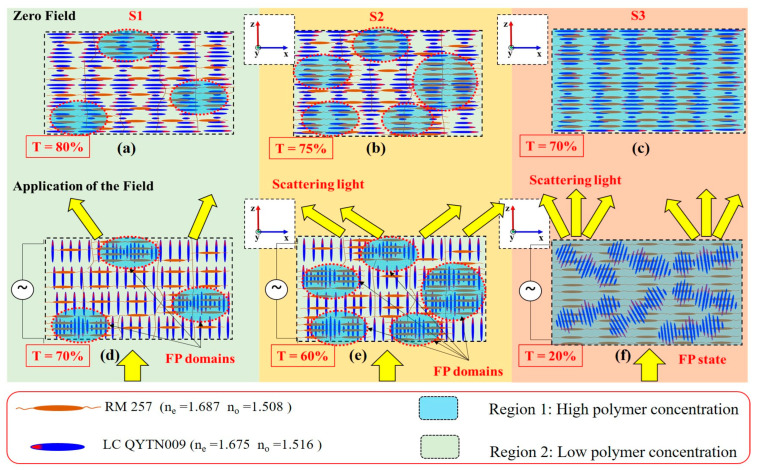
Schematic illustration describing the mechanism of light scattering for S1, S2, and S3 at zero field (**a**–**c**) and under E-field (**d**–**f**). T: transmission, FP: fingerprint.

Furthermore, we investigated the influence of polymer concentration on light scattering in three samples. Figure 6 illustrates a schematic description of light scattering and texture transition for S1, S2, and S3. Our results clearly indicate that increasing the polymer concentration reduces light transmission, i.e., increases light scattering. For example, at zero field, S1 exhibited 80% transmission, while in S3, the transmission decreased to 70% at the LWBE. By increasing the E-field, the transmission of S1 dropped to 60%, whereas for S3, it dropped to 20%. It is evident that the inclusion of the polymer matrix in the cholesteric structure affects the transmission of light through the cell. To simplify the discussion, we assume that the configuration leads to the formation of two kinds of regions: Region 1 (high polymer density, the blue-cyan color), and Region 2 (low polymer concentration, green color).

In the absence of an external field, S1, S2, and S3 exhibited the planar state, where the helical axes are perpendicular to the cell surfaces, as illustrated in Figure 6a–c, respectively. In sample S1, a few domains with high polymer concentrations are formed (Region 1), while more domains with high concentration are formed in S2, and even more in S3. When a threshold electric field is applied, the molecules rotate by a torque to align with the E-field. The orientation of the LC molecules depends on the electric field strength and the rigidity of the polymer matrix. For example, in S1, the torque on the LC molecules occurs at a very low electric field amplitude. The rotation of the LC molecules is accompanied by the formation of an unstable fingerprint (FP) domain located in Region 1, as in Figure 6d. These domains are observed as white and black dots in the POM micrograph, as previously shown in Figure 5a and Figure S3. These domains are responsible for the weak scattering of light in S1, due to the small domain size infiltrated with the LC molecules. For sample S2, more regions with high polymer concentration appear, causing a slight increase in light scattering compared with S1, as in Figure 6b. Consequently, more FP domains were formed, as in Figure 6e and Figure S3. At a critical polymer concentration, the FP domains grow and spread across the entire cell surface, becoming more stable, as shown in S3 (Figure 6c). These domains are marked by the undulation of the stripe pattern throughout the cell, as previously shown in the POM micrograph in Figure 5c. The growth of these domains, combined with the application of the E-field, leads to a decrease in transmitted light. In addition, the direction of the stripe pattern strongly depends on the confinement ratio *C*; in our case, *C* ≪ 1. This is why the stripe pattern exhibits a zigzag-like undulation rather than aligning parallel to the cell surfaces. This can explain the drop in transmission from 70% at zero field to around 20% at 10 V/μm, (Figure 6c,f).

To summarize, at lower polymer concentrations, there is a greater tendency for the formation of two separate domains: one with high polymer concentration (Region 1) and the other with low polymer concentration (Region 2). This can result in the formation of areas with different refractive indices, leading to increased light scattering. For S1, when an electric field is applied, the LC molecules rotate at a low E-field. Consequently, the difference in the refractive index between the separate regions is relatively low, leading to low light scattering, with an average refractive index of 1.508 ≤ *n_avg_* ≤ 1.516. For S2, the tendency for LC reorientation with the electric field is relatively low, causing a variation in the refractive index similar to S1. At higher polymer concentrations, this tendency becomes stronger. As a result, when the electric field is applied, the denser polymer network prevents the LC molecules from reorienting with the E-field, with their average refractive indices being (1.675 ≤ *n_avg_* ≤ 1.687), which leads to high light scattering. It is important to highlight that, under a strong electric field, the three PSCLC samples have the same characteristic, in which the LC molecules align with the electric field.

## 4. Dissymmetry Factor *g*(*λ*)

As predicted, the increasing of the polymer concentration in a CLC host enhanced the stability of LC molecules under the application of an E-field. To investigate the effect of polymer concentration on the stability of LC molecules, we measured the circularly polarized luminescence (CPL) for the two PSCLC samples, S1 and S3, under E-field excitation. Experimentally, 1 wt. % of PM597 and 1 wt. % of the DCM fluorescent laser dyes (both from Exciton, Dayton, OH, USA) were added to the first (S1) and the third (S3) PSCLC samples, respectively. The normalized fluorescence and absorbance spectra of both laser dyes in the nematic host are presented in Figure S4. The PBG of S1 was tuned to match the maximum fluorescence of PM597 dye, by changing the concentration of the compounds (adding a small quantity of the nematic/polymer). The dispersion of the laser dye in the PSCLC caused a slight red shifting of the PBG. Thus, the PBG of both S1 and S3 under various E-field amplitudes are illustrated in Figure S5. The circularly polarized degree (CPD) quantifies the difference between the emissions of right-handed or left-handed circularly polarized light, which is commonly characterized by the dissymmetry factor *g*(*λ*), given by [38,39]:

(1) $$g(\lambda )=2*\frac{I_{L}-I_{R}}{I_{L\;}+I_{R}}$$

where *I_L_* and *I_R_* represent the left-handed and right-handed luminescence intensities, respectively. 

**Figure 7:**
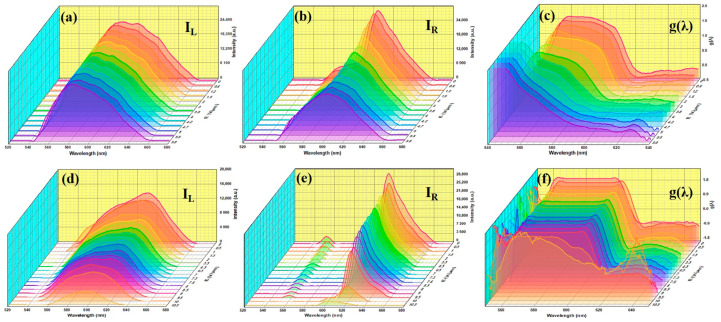
The measured right (*I_R_*) and left (*I_L_*) CP transmission spectra for S1 (**a**,**b**) and S3 (**d**,**e**), and the CPD (*g*(*λ*)) for transmitted light for S1 (**c**) and S3 (**f**) under various E-field amplitudes.

Figure 7 displays the measured right (*I_R_*) and left (*I_L_*) CPL transmission spectra for S1 and S3 under various E-field amplitudes. The left-handed circularly polarized (LCP) component experiences a significant reduction in intensity at the center of the PBG (Figure 7a,d). This reduction can be attributed to the forbidden DOS at the center of the PBG. In contrast, the right-handed circularly polarized (RCP) component resembles the emissions of the laser dye (PM597 in S1 and DCM in S3). The intensity of the RCP component is improved at the edge of the PBG and shows less intensity in the middle (Figure 7b,e). Consequently, even with the poor alignment of the laser dye within the PSCLC system, light emitted at the center of the PBG is notably circularly polarized. The increasing of the E-field resulted in a reduction in the intensity of both RCP and LCP components, along with a distortion in the shape of the RCP.

In Figure 7, we also illustrate *g*(*λ*) of transmitted light for S1 and S3, calculated using Equation (1). By comparing the *g*(*λ*) values of both S1 and S3, it is clear that the shape of the spectra for S1 was distorted at low E-field amplitudes (Figure 7c). In contrast, the spectra shape for S3 was preserved even at high E-fields (Figure 7f). As we know, developing stimuli-responsive CPL-active materials with a high CPD and effective controllability is crucial. Currently, *g*(*λ*) values of most CPL-active materials with dynamic switching capabilities range from 10^−2^ to 10^−1^, significantly below the theoretical maximum requirement [40]. Theoretically, *g*(*λ*) ≈ ±2 corresponds to pure CPL emission, with +2 indicating LCP and −2 indicating RCP [39,40,41].

**Figure 8:**
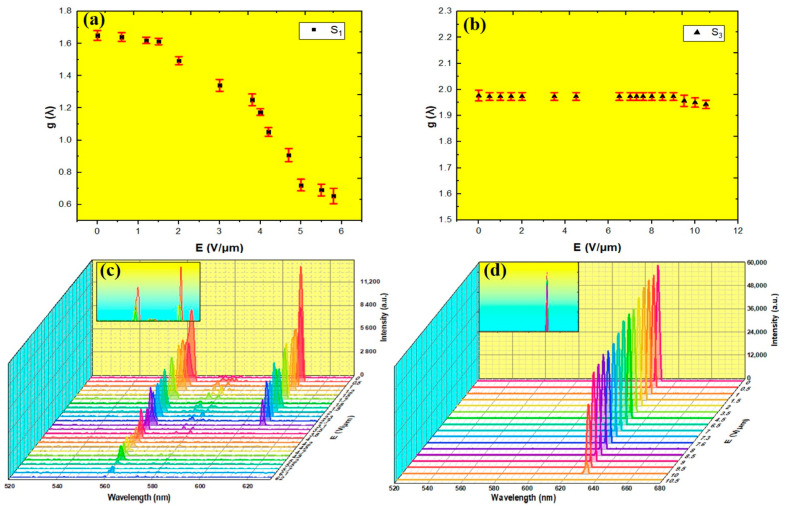
The maximum dissymmetry factor gmax (**a**) for S1, and (**b**) for S3, and LCP lasing emission spectra obtained from PM597 + S1 and DCM + S3 samples. The error bars (red bars) indicate the standard deviation. Insets in (**c**,**d**) show the positions of laser peaks.

Figure 8a,b illustrate the maximum dissymmetry factor *g_max_* at 572 nm and 580 nm for S_1_ and S3, respectively. For S1, *g_max_* decreased as the electric field increased, dropping from approximately 1.6 at zero field to 0.7 at 6 V/μm. In contrast, *g_max_* for S3 remained relatively constant, around 1.98, from zero to 11 V/μm. This difference can be attributed to the different polymer concentrations in both samples. More specifically, with a lower polymer concentration (3 wt. %), S1 has a less dense polymer network, offering weaker stabilization to LC molecules. Consequently, the LC molecules in S1 are more disposed to reorient and distort when an electric field is applied, resulting in a more pronounced distortion in the spectral shape of the CPL at lower E-field amplitudes. Conversely, S3, with a higher polymer concentration (10 wt. %), forms a denser and more robust polymer network. This stronger network better stabilizes the alignment of the LC molecules, even under higher E-field amplitudes. These findings indicate that the quality factor of the uniform standing helix was preserved during the tuning process.

For lasing experiments, the second harmonic of the Nd: YAG laser irradiated the cell in a normal direction. When the pumping beam hit sample S1, two laser lines were simultaneously observed at 664 nm and 610 nm (Figure 8c). The pumping energy was around 2.5 µJ/pulse. At zero field, the characteristic oily streak texture of planar alignment was observed as seen in Figure 5a. In this texture, the helical axis was aligned perpendicularly to the cell surfaces and the LWBE and SWBE of the PBG were located at 560 nm and 610 nm, respectively. For an unpolarized incident light, a CLC cell with a right-handed helix reflects RCP light and transmits LCP light. The transmitted light follows the helical twist of the LC molecules within the cholesteric structure. Consequently, any distortion of the helical structure can disrupt the path of transmitted light, resulting in a decrease in light intensity. When an E-field was applied, a reduction of the lasing intensity occurred. At 3.5 V/μm, the laser peak observed at the LWBE was suppressed, accompanied by a slight shift of the laser peak observed at the SWBE by ~4 nm. This is likely due to the transition from the planar state to the unstable fingerprint texture, which disturbs the helical structure.

At a high E-field, both laser lines were suppressed confirming the transition of the fingerprint texture to the homeotropic state, in which the helical structure was unwound. In contrast, when the pumping beam hit S1, a single laser line was observed at 629 nm, overlapping with the LWBE (see Figure 8d). Even at a high E-field, the lasing intensity remained stable. The pumping energy was around 3.1 µJ/pulse. This stability can be explained by the increased polymer concentration, which stabilizes the phase and conserves the helical alignment at high voltage.

## 5. Conclusions

In conclusion, we have investigated the optical properties of PSCLCs under the influence of an applied electric field. The study focused on PSCLC samples with varying polymer concentrations, providing a detailed analysis of their electro-optical characteristics. The results reveal significant shifts in the PBG of the PSCLC samples in response to the applied electric field. Specifically, the PBG exhibited complex behaviors, including blue shifts, asymmetric broadening, and contraction, depending on the polymer concentration and field strength. These phenomena were attributed to the local reorientation of the liquid crystal molecules within the polymer matrix, which affected the refractive index. Furthermore, we investigated the dissymmetry factor, which characterizes the circularly polarized luminescence of the PSCLC samples under excitation. The results demonstrate that the dissymmetry factor varied with polymer concentration and electric field amplitude, highlighting the influence of these parameters on the stability and controllability of CPL emission and lasing emission. This work contributes to a deeper understanding of the electro-optical behavior of PSCLCs and offers valuable insights for the design and optimization of these materials for applications such as displays, sensors, and photonic devices [42,43,44].

## Figures and Tables

**Table 1:** Composition of the three samples with the different weight ratios.

	CLC		PCLC		
	Nematic	Chiral Dopant	CLC	Achiral Monomer	P. Initiator
	QYTN-009	R5011	QYTN-009 + R5011	RM257	Irg651
Sample 1	97.5 wt %	2.5 wt %	96 wt %	3 wt %	1 wt %
Sample 2			93 wt %	6 wt %	1 wt %
Sample 3			89 wt %	10 wt %	1 wt %

**Table 2:** The threshold electric field at different texture transitions between fingerprint (EFP), focal conic (EFC), and homeotropic (EH) states for the four samples.

Samples	E_FP_ (V/μm)	E_FC_ (V/μm)	E_H_ (V/μm)
S0	-	0.74	1.5
S1	-	4	9
S2	-	5	10
S3	6	8 / 9	12

## Data Availability

The data that support the findings of this study are available from the corresponding author upon reasonable request.

## Supplementary Materials

The following supporting information can be downloaded at: https://www.mdpi.com/article/10.3390/polym16172436/s1, Figure S1: (a) experimental transmission spectra and (b) theoretical transmission spectra (measured with the Berreman matrix method) with the corresponding variation of the LWBE, SWBE, central wavelength, and the width of the PBG. The fitting parameters were included in the theoretical transmission spectra (b); Figure S2: (a) Recovering of the PBG after removing the E-filed recorded for the sample S3 at various times, (b) POM micrograph after field off; Figure S3: The POM micrograph of S1 (top) and S2 (bottom) when zoomed out reveals black and white dots, which indicate the formation of unstable FP domains. Some of these domains are highlighted by red circles; Figure S4: Normalized Fluorescence and absorbance spectra of PM597 and DCM in the Nematic liquid crystal host QYTN009; Figure S5: PBG of S1 (a) and S2 (b) under various E-filed amplitudes.
