# Supplementary material for: Electro-Optic Response of Polymer-Stabilized Cholesteric Liquid Crystals with Different Polymer Concentrations
Source: Polymers (Basel). 2024 Aug 28;16(17):2436. doi: 10.3390/polym16172436 (PMC11398086; doi:10.3390/polym16172436)
Supplement: Supplementary file 1 [file polymers-16-02436-s001.zip › polymers-3128858-supplementary.pdf]

Supplementary Information

Electro-optic response of Polymer-stabilized cholesteric liquid crystals with different polymer concentrations

Lotfi Saadaoui [1,2], Donghao Yang [1], Faheem Hassan [1], Ziyang Qiu [1], Yu Wang [1], Yujie Fan [1], Irena Drevensek-Olenik [3,4,*], Yigang Li [1], Xinzheng Zhang [1,5,*] and Jingjun Xu [1]

1 The MOE Key Laboratory of Weak-Light Nonlinear Photonics and International Sino-Slovenian Join Research Center on Liquid Crystal Photonics, TEDA Institute of Applied Physics and School of Physics, Nankai University, Tianjin 300457, China; lotfi.saadaoui@fst.utm.tn (L.S.); yangdonghao0305@126.com (D.Y.); faheemhassan118@hotmail.com (F.H.); 2120230309@mail.nankai.edu.cn (Z.Q.); 1120190080@mail.nankai.edu.cn (Y.W.); 2210369@mail.nankai.edu.cn (Y.F.); liyigang@nankai.edu.cn (Y.L.); jjxu@nankai.edu.cn (J.X.)

2 Physics Laboratory of Soft Matter and Electromagnetic Modelling, Faculty of Sciences of Tunis, University of Tunis El Manar, El Manar Tunis 2092, Tunisia

3 Faculty of Mathematics and Physics, University of Ljubljana, SI-1000 Ljubljana, Slovenia

4 Department of Complex Matter, J. Stefan Institute, SI-1000 Ljubljana, Slovenia

5 Collaborative Innovation Center of Extreme Optics, Shanxi University, Taiyuan 030006, China

* Correspondence: irena.drevensek@ijs.si (I.D.-O.); zxz@nankai.edu.cn (X.Z.)

(a) **(b)**

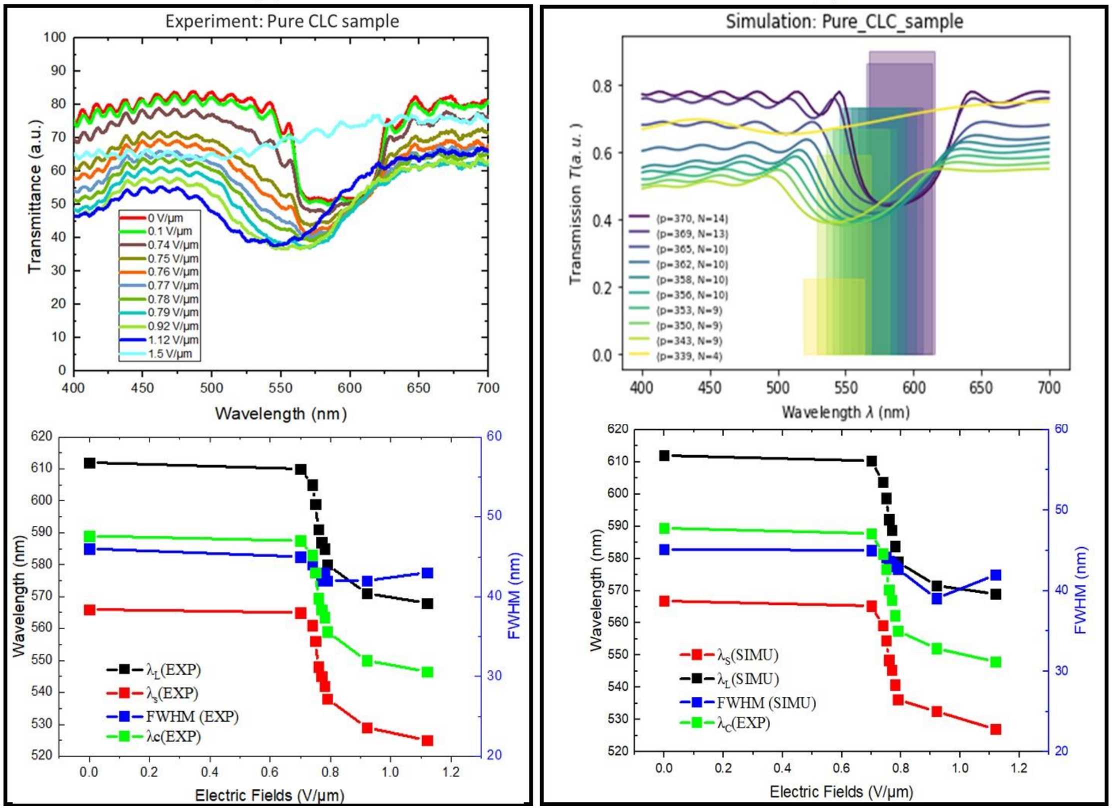

Figure S1: (a) experimental transmission spectra and (b) theoretical transmission spectra (measured with the Berreman matrix method) with the corresponding variation of the LWBE,

SWBE, central wavelength, and the width of the PBG. The fitting parameters were included in the theoretical transmission spectra (b).

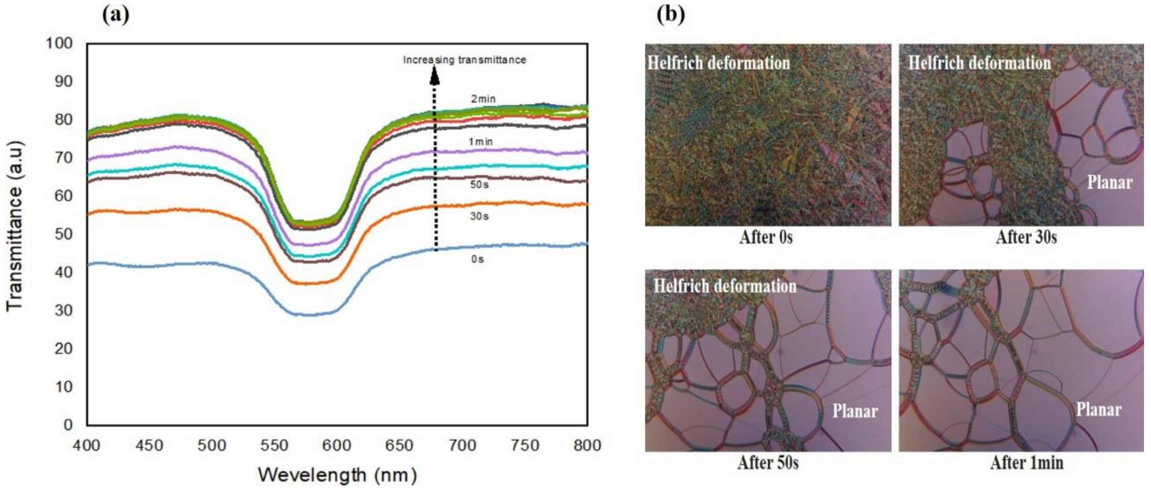

Figure S2: (a) Recovering of the PBG after removing the E-filed recorded for the sample S3 at various times, **(b)** POM micrograph after field off

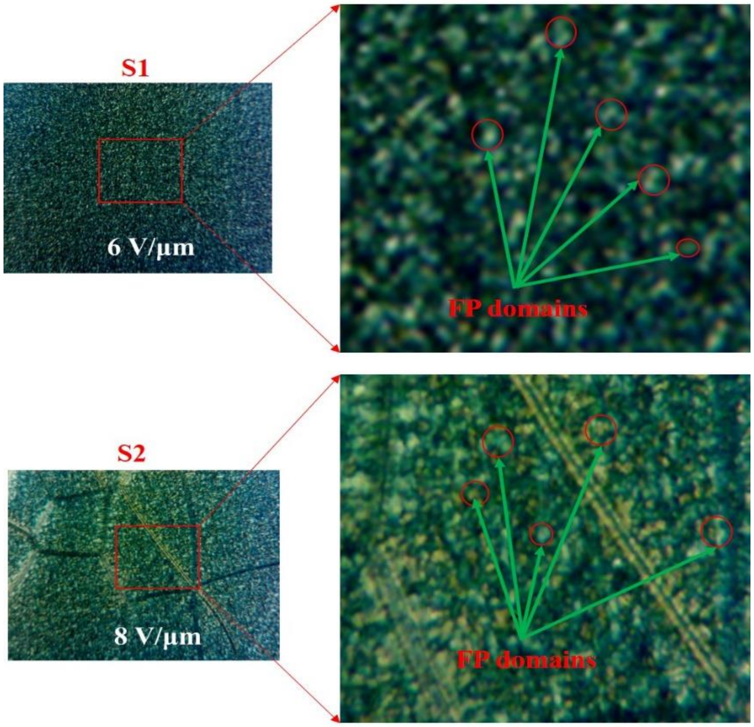

Figure S3: The POM micrograph of S1 (top) and S2 (bottom) when zoomed out reveals black and white dots, which indicate the formation of unstable FP domains. Some of these domains are highlighted by red circles.

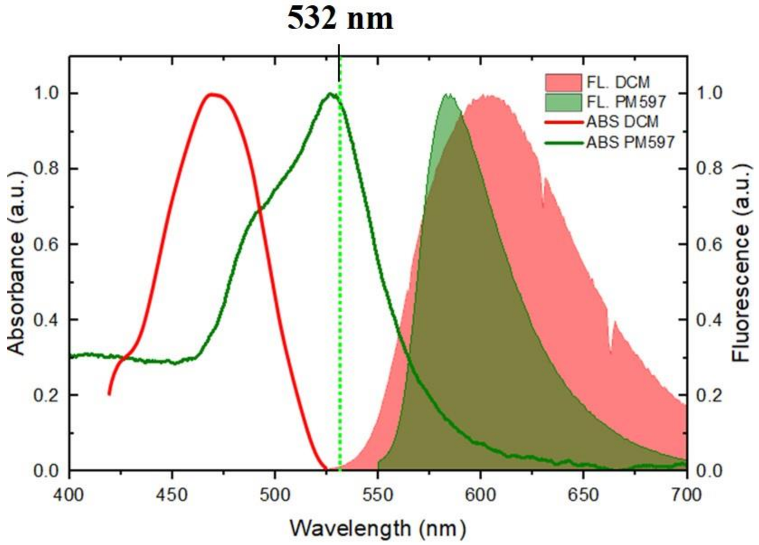

Figure S4: Normalized Fluorescence and absorbance spectra of PM597 and DCM in the Nematic liquid crystal host QYTN009.

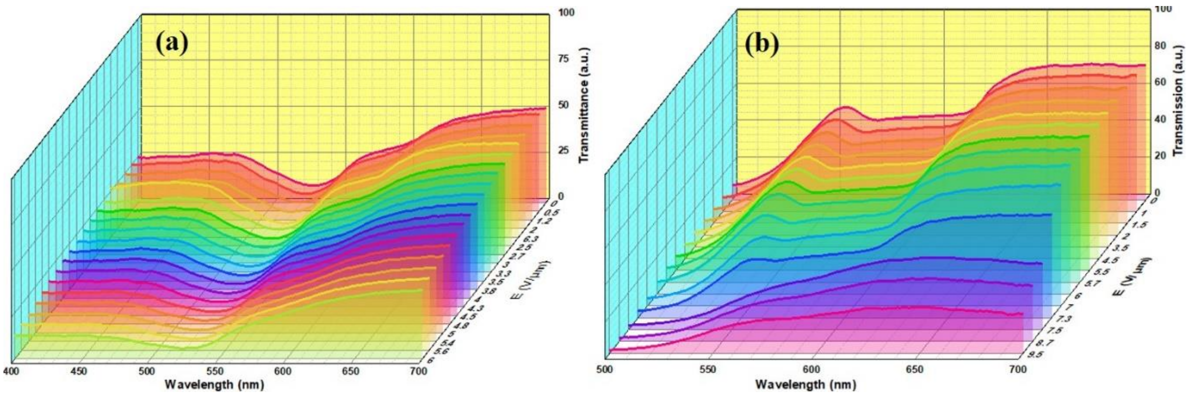

Figure S5: PBG of S_1 (a) and S_2 (b) under various E-filed amplitudes.
